# Public health impact of foodborne exposure to naturally occurring virulence-attenuated *Listeria monocytogenes:* inference from mouse and mathematical models

**DOI:** 10.1098/rsfs.2019.0046

**Published:** 2019-12-13

**Authors:** Alison Stout, Anna Van Stelten-Carlson, Hélène Marquis, Michael Ballou, Brian Reilly, Guy H. Loneragan, Kendra Nightingale, Renata Ivanek

**Affiliations:** 1 Department of Population Medicine and Diagnostic Sciences, Cornell University, Ithaca, NY, USA; 2 Department of Microbiology and Immunology, College of Veterinary Medicine, Cornell University, Ithaca, NY, USA; 3 Department of Animal and Food Sciences, Texas Tech University, Lubbock, TX, USA; 4 Department of Veterinary Sciences, Texas Tech University, Lubbock, TX, USA; 5 Department of Biological Sciences, Texas Tech University, Lubbock, TX, USA; 6 Department of Immunology and Microbiology, Texas Tech University Health Sciences Center, Lubbock, TX, USA; 7 School of Veterinary Medicine, Texas Tech University, Amarillo, TX, USA

**Keywords:** *Listeria monocytogenes*, listeriosis, *inlA*, mathematical modelling, foodborne exposure, immune boosting

## Abstract

Listeriosis is a clinically severe foodborne disease caused by *Listeria monocytogenes* (Lm). However, approximately 45% of Lm isolates in food carry a virulence-attenuating single-nucleotide polymorphism in *inlA*, which normally facilitates crossing the intestinal barrier during the initial stages of infection. We hypothesized that (i) natural exposure to virulence-attenuated (vA) Lm strains through food can confer protective immunity against listeriosis attributable to fully virulent (fV) strains and (ii) current food safety measures to minimize exposure to both Lm strains may have adverse population-level outcomes. To test these hypotheses, we evaluated the host response to Lm in a mouse infection model and through mathematical modelling in a human population. After oral immunization with a murinized vA Lm strain, we demonstrated the elicitation of a CD8+ T-cell response and protection against subsequent challenge with an fV strain. A two-strain compartmental mathematical model of human exposure to Lm with cross-protective immunity was also developed. If food safety testing strategies preferentially identify and remove food contaminated by vA strains (potentially due to their common occurrence in foods and higher concentration in food compared to fV strains), the model predicted minimal public health benefit to potentially adverse effects. For example, reducing vA exposures by half, while maintaining fV exposures results in an approximately 6% rise in annual incidence.

## 1. Introduction

*Listeria monocytogenes* (Lm) is a facultatively intracellular foodborne pathogen and the causative agent of a potentially life-threatening systemic disease known as listeriosis [1]. Clinical manifestations of listeriosis include septicaemia, encephalitis, meningitis and late-term spontaneous abortions or stillbirths in pregnant women [2]. Lm infections are associated with notably high hospitalization (94%) and case-fatality rates (16%), accounting for approximately 19% of all fatalities attributed to known foodborne pathogens each year in the USA [3]. Foodborne transmission, and in particular ready-to-eat (RTE) foods as a vehicle of exposure, provoked establishment of a ‘zero tolerance’ policy in the USA for detection of Lm in RTE foods [4]. The 2003 Lm risk assessment for the USA predicted that the average American consumes food servings containing low (less than 1 × 10^3^ microorganisms) or intermediate (between 1 × 10^3^ and 1 × 10^6^ microorganisms) levels of Lm 19 and 2.4 times per year, respectively [5]. Consumption of a high (>1 × 10^6^ microorganisms) level of Lm occurs approximately once every 15 months [5]. Thus, the average American consumes Lm approximately 22 times per year.

The 2003 Lm risk assessment identified RTE deli meats as the most common food responsible for listeriosis (approx. 90% of cases) [5]. However, declines in the incidence of listeriosis have not paralleled reductions in the prevalence of Lm-contaminated deli meats, which has been reduced by more than ninefold (from 2.54% in 1998 to 0.28% in 2010), while the comparative incidence of listeriosis was not even reduced by half (from 5 cases/1 000 000 in 1996 to 2.6 cases/1 000 000 in 2012) [6,7]. Of note, the incidence of listeriosis has increased in parts of Europe [8]. Changing consumer demographics and eating habits could in part explain the public health trends. However, in this study, we aim to evaluate whether the trends could be explained by a reduction in exposure to less virulent Lm strains that may confer protective immunity. Under such a mechanism, a total reduction in Lm exposures (i.e. both strains are decreased proportionately) could result in a slower decline in listeriosis cases than expected for the achieved reduction in exposures.

Lm is genetically diverse and significant evidence supports the presence of two epidemiologically distinct subpopulations in food, including (i) fully virulent (fV) strains that have been linked to the majority of sporadic and epidemic listeriosis cases and (ii) virulence-attenuated (vA) strains that represent greater than 45% of isolates from food but have only been linked to sporadic disease on very rare occasions (approx. 5%) [9]. In addition, at retail, vA Lm subtypes are present at higher concentrations in contaminated RTE food when compared with fV strains (2 × 10^5^ versus 1.76 × 10^3^ for vA and fV, respectively) [10]. Virulence attenuation of these isolates is attributed to specific single-nucleotide polymorphisms (SNPs) in the key virulence gene *inlA*, ultimately leading to a premature stop codon (PMSC)*.* The mechanism of virulence attenuation by these SNPs is production of a truncated and secreted gene product (InlA) or loss of InlA production, and thus, virulence attenuation by preventing effective transfer across the intestinal barrier during the initial stages of an infection [11].

While it has been shown previously that exposure to an Lm strain carrying a vA *inlA* PMSC at a high dose (1 × 10^10^ colony forming units (CFU)) confers protection against subsequent challenge (15 days post-infection) by an fV strain in a guinea pig model [12], the need remains to determine if protection can be provided by vA strains administered at levels representative of contamination commonly found in food. Essentially, vA contaminations in food may have the ability to act as natural vaccines, eliciting an immune boost and providing protection against more deleterious exposures to fV Lm. The overall goal of this study was to investigate whether exposure to a vA Lm strain has a protective effect against listeriosis and we investigated this at the individual host and population levels, respectively, using a combination of mouse infection experiments and mathematical modelling of a human population.

## 2. Methods and material

### 2.1. Experimental mouse model

#### 2.1.1. Oral mouse infection

The immune response to challenge by Lm was evaluated in female BALB/c mice (8–10 weeks old; Charles River Laboratories, North Wilmington, MA, USA), housed in individual cages at the Texas Tech University Laboratory Animal Resources Facility adhering to regulations outlined by the Institutional Animal Care and Use Committee (Animal protocol T12002). Animals were acclimated for a minimum of 5 days and provided feed and water ad libitum. Table 1 lists *Listeria* strains used in mouse experiments. Through a feeding needle, animals were compelled to ingest a dose of one of four *Listeria* strains in table 1 as a treatment resuspended in a 100 µl volume supplemented with calcium carbonate (CaCO_3_; 100 mg ml^−1^) or a placebo carrier solution of phosphate-buffered saline (PBS) to represent. The feeding needle was flushed with 100 µl of PBS to ensure full inoculum delivery. A minimum of three animals (biological replicates) were exposed to carrier solution or each *Listeria* strain at 2 × 10^3^, 2 × 10^5^ or 2 × 10^7^ CFU to simulate low, intermediate and high exposure levels in the 2003 Lm risk assessment resulting in (i) eight treatment groups in the immune response experiments in which CD 8+ T-cell immune response was evaluated (table 2) and (ii) seven treatment groups in the vaccine challenge experiments in which Lm recovery from internal organs was evaluated (table 3). All primary challenges ranged between 2 × 10^3^ and 2 × 10^7^ which capture levels that could foreseeably be naturally consumed [5]. All animals were examined for listeriosis symptoms and weighed daily. Fresh feed and water were provided daily and cages cleaned twice weekly; inoculated animals were not comingled.

**Table 1.** Description of Lm strains used for cell culture and animal infection experiments.

strain (previous name)	genotype or description	reference or source
*L. monocytogenes* EGD-e	fully virulent laboratory control strain encoding a full-length InlA protein	[13]
*L. monocytogenes* EGD-eM* (EGD-eInlA^m*^)	a murinized form of EGD-e; constructed in the background of EGD-e by substituting three nucleotides to increase affinity for the murine isoform of E-cadherin	[14]
*L. monocytogenes* EGD-eM*: PMSC3	EGD-eM* carrying a virulence-attenuating SNP leading to PMSC mutation 3	this study
*L. innocua* TTU B1-019 (ATCC 33090)	ATCC *Listeria innocua* control strain	American Type Culture Collection (ATCC) (Manassas, VA, USA)

**Table 2.** Treatment groups and strain–dose combinations in immune boosting experiments to evaluate the recall of the CD8+ T-cell immune responses. PBS, phosphate buffer solution; n.a., not applicable; Li, *Listeria innocua*; Lm, *Listeria monocytogenes;* fV, fully virulent; vA, virulence attenuated.

	primary challenge			secondary (booster) challenge	
group	description	strain	dose (CFU)	strain	dose (CFU)
unvaccinated	negative control (PBS)	placebo	n.a.	Lm EGD-eM*	2 × 10^7^
vaccinated	non-pathogenic control	Li	2 × 10^7^	Li	2 × 10^7^
vaccinated	non-pathogenic control	Li	2 × 10^5^	Li	2 × 10^5^
vaccinated	non-pathogenic control	Li	2 × 10^3^	Li	2 × 10^3^
vaccinated	dose common in foods for vA strain^a^	Lm EGD-eM*:PMSC3	2 × 10^5^	Lm EGD-eM*:PMSC3	2 × 10^5^
vaccinated	dose common in foods for fV strain^a^	Lm EGD-eM*	2 × 10^3^	Lm EGD-eM*	2 × 10^3^
vaccinated	high dose^a^	Lm EGD-eM*:PMSC3	2 × 10^7^	Lm EGD-eM*:PMSC3	2 × 10^7^
vaccinated	high dose^a^	Lm EGD-eM*	2 × 10^7^	Lm EGD-eM*	2 × 10^7^

^a^Doses correspond to classification levels previously defined in the 2003 risk assessment (FDA/USDA/CDC, 2003) and doses common in foods are inferred from Chen *et al*. [10].

**Table 3.** Treatment groups and strain–dose combinations in vaccine challenge experiments to evaluate the protective immunity induced by *Listeria* spp. exposure. PBS, phosphate buffer solution; n.a., not applicable; Li, *Listeria innocua*; Lm, *L. monocytogenes*; fV, fully virulent; vA, virulence attenuated.

	primary challenge			secondary challenge	
group	description	strain	dose (CFU)	strain	dose (CFU)
unvaccinated	negative control (PBS)	placebo	n.a.	Lm EGD-eM*	2 × 10^7^
vaccinated	non-pathogenic control	Li	2 × 10^7^	Lm EGD-eM*	2 × 10^7^
vaccinated	dose common in foods for fV strain^a^	Lm EGD-eM*	2 × 10^3^	Lm EGD-eM*	2 × 10^7^
vaccinated	dose common in foods for vA strain^a^	Lm EGD-eM*:PMSC3	2 × 10^5^	Lm EGD-eM*	2 × 10^7^
vaccinated	intermediate dose^a^	Lm EGD-eM*	2 × 10^5^	Lm EGD-eM*	2 × 10^7^
vaccinated	high dose^a^	Lm EGD-eM*:PMSC3	2 × 10^7^	Lm EGD-eM*	2 × 10^7^
vaccinated	high dose^a^	Lm EGD-eM*	2 × 10^7^	Lm EGD-eM*	2 × 10^7^

^a^Doses correspond to classification levels previously defined in the 2003 risk assessment (FDA/USDA/CDC, 2003) and doses common in foods are inferred from Chen *et al*. [10].

Each treatment group was maintained for greater than 40 days post-inoculation, the time required for T-cell conversion to a central memory phenotype following primary Lm infection [15]. Post-inoculation, animals received a secondary challenge. Mice allocated for the CD8+ T-cell ‘immune boosting’ experiment were re-challenged 72 h prior to euthanasia with the same strain–dose combination used at primary inoculation; PBS-negative control mice were challenged with EGD-eM* at a high dose (2 × 10^7^ CFU) (table 2). To evaluate protective immunity against challenge with an fV Lm strain, in a separate ‘vaccine challenge’ experiment, mice were re-challenged with EGD-eM* at a high dose (2 × 10^7^ CFU) 72 h prior to euthanasia (table 3). In that vaccine challenge experiment, recovery levels (in log_10_ CFU g^−1^) of Lm from the spleen, liver and small intestine were used as the main measure of protection provided by vaccination (i.e. strain–dose treatment). According to existing Lm risk assessments based on non-pregnant animal challenge studies, recovery of Lm from the spleens was considered the organ most indicative of systemic Lm infection [5,16]. Bacterial counts for organ samples that were either negative by direct plating but positive by enrichment or negative by both direct plating and enrichment were reported as the detection limit of direct plating or the detection limit of enrichment, respectively. The frequency of antigen-specific CD8+ T cells induced in mouse spleens greater than 40 days after primary infection was determined through evaluation of the number of IFN-γ producing CD8+ T cells induced at re-challenge with the same strain–dose combination as at the primary challenge, showing reactivity against the dominant LLO_91–99_ epitope.

#### 2.1.2. Statistical analysis of mouse infection experiments

All statistical analysis was performed using a linear mixed model as implemented in the MIXED procedure of SAS (Statistical Analysis Systems Software, Cary, NC, USA). For the vaccine challenge experiment observing bacterial counts, a factorial one-way analysis of variance (ANOVA) was used, where replicate was the random effect, and strain–dose combination and organ were fixed effects. For the experiment observing IFN-γ frequency, a nested ANOVA was used with nesting within the treatment (vaccinated versus unvaccinated) groups. Replicate was included as a random effect, while dose, strain and treatment were all fixed effects in the model. The mean separation on independent variables, for which significant treatment effects were identified, was performed using an lsmeans statement. Log_10_ transformations were used where appropriate; a Shapiro–Wilk's test for normality and Levene's test for homogeneity were run to ensure data met assumptions of the ANOVA. Probabilities of less than 0.05 were considered statistically significant.

Additional methods for bacterial strain characterization and mouse infection studies are described in the electronic supplementary material.

## 3. Mathematical modelling

### 3.1. Model set-up

**Figure 1.**
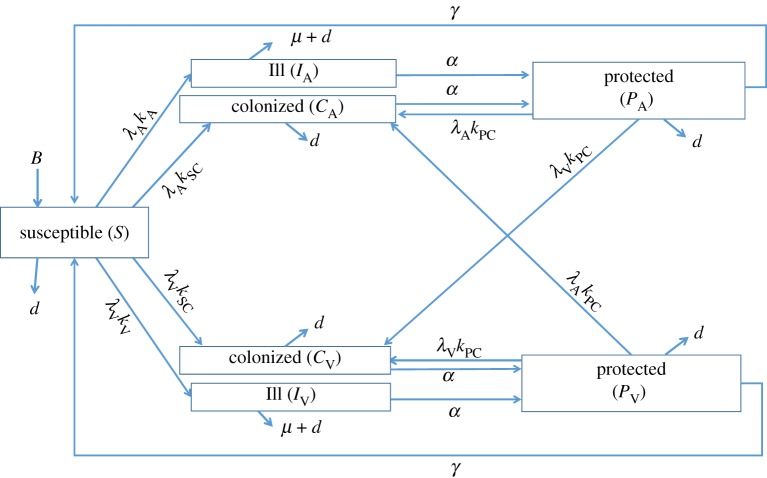
A diagram of the developed mathematical model of human exposure to Lm through foods and immune boosting. Parameters are defined in table 4. (Online version in colour.)

A two-strain model of Lm exposures and cross-protection was built using a system of ordinary differential equations (see electronic supplementary material). The model is presented in figure 1 and associated parameters are given in tables 4 and 5. Briefly, the model captures the presence of two naturally occurring Lm subpopulation strains, vA and fV (denoted with subscript A and V, respectively, in equations and parameter notations). From the Susceptible compartment, *S*, for each given strain, there are two possible states post exposure: either the Ill compartment, *I*, representing clinical illness or the Colonized compartment, *C*, representing an intermediate immune state with no clinical disease, essentially an asymptomatic infection state. After being in the Ill or Colonized compartment for a strain, individuals recover into the Protected compartment, *P*, for the strain at a rate *α*, where it is assumed that protective immunity against clinical disease is present. While protected, additional exposures to either vA or fV Lm can result in a return to colonization, with subsequent transition into the *P* compartment, where a repeated immune response is assumed. In the absence of re-exposures to Lm while in compartment *P*, immunity wanes and protected individuals revert to the Susceptible compartment at a rate *γ*. The success of Lm foodborne exposure is defined by the rate of exposure (*λ*) and the probability of infection or colonization given exposure (*κ*), which are further characterized below. If exposure was not successful, there is no change in status. Individuals enter the population through the susceptible compartment via births (*B*). Natural deaths occur at rate *d*, while listeriosis deaths from the Ill compartments occur at rate *μ*. The model describes the US human population, with the constant total population size, *N*, set to 3 × 10^8^, where

3.1 $$\begin{array}{r} N(t)=S(t)+I_{A}(t)+C_{A}(t)+I_{V}(t)+C_{V}(t)+P_{A}(t)+P_{V}(t). \end{array}$$

**Table 4.** Definitions of model parameters and their baseline values. Lm, *Listeria monocytogenes*; n.a., not applicable.

description	parameter	value	unit	reference
US population	*N*	3 × 10^8^	people	[17]
total number of foodborne exposures to Lm	*E*_t_	22	exposures/person/year	[5]
proportion of total foodborne exposures to Lm attributed to a virulence-attenuated strain	pA	0.45	n.a.	[9]
number of foodborne exposures to virulence-attenuated Lm; estimated as (pAE_t_)	*E*_A_	10	exposures/person/year	[9]
number of foodborne exposures to fully virulent Lm; estimated as [(1 − pA)E_t_]	*E*_V_	12	exposures/person/year	[9]
rate of exposure to virulence-attenuated strain	*λ*_A_	0.0278	day^−1^	[5]
rate of exposure to fully virulent strain	*λ*_V_	0.0334	day^−1^	[5]
probability of clinical disease given exposure to virulence-attenuated strain	$\kappa_{A}$	3.48 × 10^−8^	n.a.	[10]
probability of clinical disease given exposure to fully virulent strain	$\kappa_{V}$	6.39 × 10^−7^	n.a.	[10]
probability of colonization by either strain for an individual in the susceptible compartment	$\kappa_{\mathrm{SC}}$	varied^a^	n.a.	estimated by Monte Carlo simulation
probability of colonization by either strain for an individual in the protected compartment	$\kappa_{\mathrm{PC}}$	varied^a^	n.a.	estimated by Monte Carlo simulation
rate of recovery from illness or colonization by either strain	*α*	1/14	day^−1^	[18]
rate of immunity loss	*γ*	varied^a^	day^−1^	estimated by Monte Carlo simulation
death rate due to listeriosis	*μ*	0.0114	day^−1^	[3]
number of births	*B*	N/(70 × 365)	people/day	[17,19]
natural death rate	*d*	1/(70 × 365)	day^−1^	[17,19]

^a^The parameter was estimated through Monte Carlo simulation as shown in table 5.

**Table 5.** Mean parameter values for *κ*_SC_, *κ*_PC_ and *γ* estimated through Monte Carlo simulation under two different calibration criteria. Parameters *κ*_SC_, *κ*_PC_ and *γ* are defined in table 4.

calibration criterion	*κ*_SC_	*κ*_PC_	*γ*
wide: 557–3161 listeriosis cases annually	0.0989	0.0992	0.0359
narrow: 1500–1700 listeriosis cases annually	0.144	0.146	0.0268

### 3.2. Model assumptions

(1) The model initial condition was set to *N*(0) = *S*(0). This was necessary because the true prevalence of individuals in different model compartments is unknown. All model predictions were interpreted at the steady state and because the model converged to the same steady state irrespective of the initial conditions, this assumption did not affect the study findings.
(2) The general US population was modelled, thereby assuming the same epidemiological characteristics with respect to Lm for the whole population. This assumption was deemed necessary because of the lack of information about strain-specific parameter values for subsets of the population (e.g. elderly, children, pregnant women and healthy adults).
(3) A constant human population size was assumed and implemented by setting the number of births into the Susceptible compartment being equal to the number of deaths due to any cause and due to listeriosis, i.e.
  3.2 $$B=dN+\mu (I_{A}+I_{V}).$$
  This improved tractability of the model; considering that the number of listeriosis deaths is very small compared to the population size, this assumption did not affect the model predictions. We also neglected immigration and emigration in the US population because of the lack of information with respect to the Lm epidemiology.
(4) Individuals colonized with Lm were assumed to be infected but clinically healthy. This assumption was deemed reasonable considering that healthy people have been reported to shed Lm in faeces [20,21].
(5) The rate of recovery from clinical illness or colonization (*α*), immunity loss (*γ*) and the natural death rate (*d*) were assumed to be exponentially distributed with the rate equal to the reciprocal of the average length of time spent in the corresponding compartment. This is a common assumption in mathematical modelling of infectious diseases and is considered to reasonably represent the disease epidemiology [22].
(6) The rate (*α*) of recovery from illness or colonization was assumed to be the same, across both strains, given limited information on these parameter values. Likewise, the loss of immunity (*γ*) was assumed to be the same for each strain.
(7) The model is simplified in regard to strain exposure in that for a given exposure to a contaminated meal, it is assumed to be to only one strain of Lm. Previous work to understand the dose–response between the two strains has also made this assumption [10].
(8) The model assumes that consumers are exposed to the same average dose of Lm of a particular strain at each exposure due to insufficient data. Although data exist about exposure levels and dose–responses for clinical illness due to vA and fV strains [10], there is lack of corresponding data about the probability of colonization, which was also one of the unidentifiable parameters in the model.
(9) The model assumes that a successfully exposed individual in the Protected compartment cannot develop a clinical illness (i.e. move into the Ill compartment). This is supported by the corresponding mouse data presented here.
(10) To evaluate implications of the assumed immune boosting in the above described model, we compared its predictions to a model without the Colonized compartments, assuming that the Colonized compartment either (i) does not exist or (ii) exists but does not affect the occurrence of clinical illness, such that the movement between the only relevant compartments is from S → I → P → S for either strain.

### 3.3. Model parameters

Based on 22 exposures (*E_t_*) per year per American [5] and the breakdown that 45% of contaminations are vA and 55% are fV [9], it is expected that 10 of those exposures would be with a vA strain (*E*_A_) and the remaining with an fV strain (*E*_V_), thus *E*_A_ = 10 and *E*_V_ = 12. Thus, the probability of exposure to a given strain per day is

3.3 $$\mathrm{vA}\;\mathrm{strain}:\;\frac{E_{A}}{365\;\;\mathrm{days}}\;$$

and

3.4 $$\mathrm{fV}\;\mathrm{strain}:\;\frac{E_{V}}{365\;\;\mathrm{days}}.$$

These daily probabilities of exposure to vA and fV strains were converted to the corresponding rates of exposure

3.5 $$\lambda_{A}=\;-\ln\left(1-\frac{E_{A}}{365}\right)=\;0.0278$$

and

3.6 $$\lambda_{V}=\;-\ln\left(1-\frac{E_{V}}{365}\right)=\;0.0334.$$

Once exposed (a contaminated meal is consumed) to either strain, susceptible individuals may develop clinical illness, with probabilities *κ*_A_ and *κ*_V_ for vA and fV strains, respectively. The probabilities of developing illness were calculated based on the exponential dose–response model for Lm infection [10], where *r* is the probability of a single cell causing disease and *D* is the dose that is consumed:

3.7 $$\mathrm{illness}\;\;\mathrm{probability}=1-\;e^{-rD}.$$

The log_10_*r* values have previously been calculated as −13.76 and −10.44 for vA and fV strains, respectively [10]. The average dose of Lm was set as 1.76 × 10^4^ CFU/serving for the fV strain and 2 × 10^6^ CFU/serving for the vA strain, which are 1-log increased from contamination levels commonly found in foods at retail [10] after accounting for an assumed increase between retail and consumption [23]. Using equation (3.5), the calculated probability of clinical illness, given exposure due to vA strain (*κ*_A_), was 3.48 × 10^−8^ and due to fV strain (*κ*_V_) was 6.39 × 10^−7^.

Upon exposure, individuals in the Susceptible or Protected compartments may become colonized with either strain with probability $\kappa_{\mathrm{SC}}$ and $\kappa_{\mathrm{PC}}$, respectively. The individuals in the Protected compartment lose the protective immunity and revert to the Susceptible compartment at a rate *γ*. The values of parameters $\kappa_{\mathrm{SC}}$, $\kappa_{\mathrm{PC}}$ and *γ* were unknown and the parameters were unidentifiable (meaning that the same model predictions could be obtained by different combinations of those parameters). Therefore, their values were simultaneously estimated through calibration using Monte Carlo simulation. A total of 10 000 iterations were conducted by selecting random values from probability distributions (described in electronic supplementary material), while all other parameter values were fixed at their values in table 4. Two calibration criteria were considered for the current annual number of listeriosis cases. ‘Wide’ corresponded with the previously reported 90% credible interval of 557–3161 for the annual number of human cases [3]. To capture the parameter space for the three parameters more closely corresponding to the current estimate for the annual number of cases, a ‘Narrow’ calibration was also used, with criteria being between 1500 and 1700 cases, centred around the estimate of nearly 1600 cases per year [3]. The subset of iterations that fit each of the two criteria for the current listeriosis cases was then analysed and the mean values for $\kappa_{\mathrm{SC}}$, $\kappa_{\mathrm{PC}}$ and *γ* in these iterations were accepted as the criterion-specific baseline values for the model (table 5) and resulted in two sets of plausible predictions. All combinations of $\kappa_{\mathrm{SC}}$, $\kappa_{\mathrm{PC}}$ and *γ* values matching each calibration criterion are shown in electronic supplementary material, figure S4.

The length of clinical illness is known to vary from a few days to a few weeks and was therefore set to the average of 14 days [16] and was considered to be the same for both strains. The length of colonization was assumed to be equal to the length of clinical illness. A natural death rate, *d*, which can occur from any compartment, was estimated based on the life expectancy of 70 years (i.e. d=1/(70×365)) [19]. For those who are clinically ill, there is a listeriosis-induced death rate, given by *μ*, which was calculated as the following, based on 16% case fatality, occurring over 14 days:

3.8 $$\mu =-\ln\left(1-\frac{0.16}{14}\right)=0.0114.$$

### 3.4. Model analysis

The time step of the model was 1 day and the model was numerically solved for 10 000 days. To interpret results, the model predictions were converted into the annual number of listeriosis cases at the model steady state. Cases were reported irrespective of whether they were attributed to the fV or vA strain. The outcome of interest was thus the cumulative number of listeriosis cases per year. Annual incidence was evaluated in year 5, by which time the model converged to a steady state in the baseline model and all tested scenarios. The model was developed in R Studio v. 1.1.453 (Boston, MA, USA) and solved using the deSolve package.

### 3.5. Model validation

The model was internally validated by comparing predictions to the previously described epidemiological data on proportion of listeriosis cases attributed to fV strain [9], case fatality [3] and prevalence of healthy people shedding Lm in faeces [20,21]. External validation was performed by applying the model to the Canadian and the European Union (EU) populations and in both cases assuming the same probabilities of food contamination with vA and fV strains as in the USA (i.e. 45% and 55%, respectively) due to lack of country-specific information. The number of births per day (*B*) is population dependent and was thus updated for the Canadian and EU populations. The Canadian population was assumed to be exposed to Lm in foods 22 times per year, i.e. at the same frequency as the US population; this was considered as a reasonable assumption, given the extent of food trade between the two countries and similar food safety standards. The population in Canada was set as 35.44 million people, corresponding to the 2014 census and the model prediction was compared to the 90% probable interval of 134–312 for the annual number of listeriosis cases [24]. In the EU, the number of annual exposures was set as 38, based on previously reported food contamination prevalences [25] and the population was set as 500 million. The total reported listeriosis cases of 2194 [26] was adjusted for underreporting using a factor of 1.7 [25]. In addition to considering the EU as one entity in the validation, we also estimated model predictions for individual member countries and used the Wilcoxon signed-rank test to compare model predictions to the country-specific numbers of reported cases adjusted for underreporting, assuming the same consumption pattern in all countries (see electronic supplementary material, table S2).

Additional information on parameters, model equations and model development is provided in the electronic supplementary material.

### 3.6. Sensitivity analysis

Each individual model parameter in tables 4 and 5 was evaluated one at a time by comparing predictions from the baseline parameter value to predictions when the baseline parameter value was increased by 1.5 or decreased by 0.5.

### 3.7. Application of the mathematical model to public health scenarios

#### 3.7.1. Proportional change

The model was used to evaluate the annual number of human listeriosis cases in the USA when the total number of exposures to contaminated foods (*E*_t_) are proportionally changed from the baseline of *E*_t_ = 22 exposures. Across all scenarios, 45% of contaminations are from the vA strain and 55% from the fV strain meaning that the ratio of strains remains the same, although *E*_t_ is changing. These scenarios represent a situation when food safety measures effectively target and remove both subpopulations proportionally.

#### 3.7.2. Single, vA or fV, strain change

Each strain was individually considered in the model. Holding the number of annual exposures to the fV strain constant (i.e. *E*_V_ = 12 to represent the baseline level or at 0, 2 and 5 annual exposures), different numbers of exposures to the vA strain were evaluated. Likewise, the opposite was considered, such that the annual exposures from the vA strain were held constant (i.e. *E*_A_ = 10 to represent the baseline level or at 0, 2 and 5 annual exposures) and the number of exposures to fV strain was varied. These scenarios represent a situation when food safety testing strategies preferentially identify and remove food contaminated by one of the strains.

## 4. Results

### 4.1. Experimental mouse results

Mice infected with EGD-eM* showed significantly higher (*p* *<* 0.05) bacterial levels in the liver, spleen, mesenteric lymph nodes and small intestines when compared with EGD-e at 72 h post-infection. EGD-eM*:PMSC3 only displayed attenuated virulence, as determined by lower bacterial loads, in livers, spleens and mesenteric lymph nodes, but not in small intestines (see electronic supplementary material, figure S2). Immune mice generated equivalent CD8+ T-cell responses following inoculation with fV and vA murinized Lm. Both the fV strain EGD-eM* and the vA strain EGD-eM*:PMSC3 were able to elicit LLO_91-99_-specific CD8+ T cells at equal frequencies (*p* > 0.05), when inoculated with 2 × 10^7^ CFU during primary infection. Specifically, total IFN-γ frequencies of 3.6% and 4.1% for EGD-eM* and EGD-eM*PMSC3 were observed, respectively (see electronic supplementary material, figure S3). Both strains elicited LLO_91-99_-specific CD8+ T cells when inoculated at their respective doses commonly found in foods at low and intermediate doses according to the 2003 risk assessment [5] (i.e. EGD-eM* at 2 × 10^3^ CFU and EGD-eM*:PMSC3 at 2 × 10^5^ CFU) at frequencies significantly higher (*p* < 0.05) than the PBS control (see electronic supplementary material, figure S3). Administration of PBS did not mount a *Listeria*-specific CD8+ T-cell response. Although animals inoculated with *L. innocua* at 2 × 10^3^ CFU and 2 × 10^5^ CFU showed no significant difference (*p* > 0.05) in total IFN-γ produced from the PBS-negative control, interestingly, animals inoculated with *L. innocua* at 2 × 10^7^ CFU mounted an IFN-γ+ CD8+ T-cell response similarly to animals infected with EGD-eM* at 2 × 10^3^ and 2 × 10^7^ CFU and EGD-eM*:PMSC3 at 2 × 10^5^ and 2 × 10^7^ CFU (see electronic supplementary material, figure S3).

Mice immunized with fV and vA murinized Lm produce significant central memory, but not effector memory, CD8+ T-cell populations in the spleen. Overall, similar trends for central memory IFN-γ+ cells were observed as in total IFN-γ for the CD8+ T-cell population. Specifically, both the fV strain EGD-eM* and the vA strain EGD-eM*:PMSC3 were able to elicit LLO_91-99_-specific CD8+ T cells at equal frequencies (*p* > 0.05) at all doses examined and at frequencies significantly higher (*p* < 0.05) than the PBS-negative control (see electronic supplementary material, figure S4). Administration with PBS did not elicit a *Listeria*-specific CD8+ T-cell response, and again, animals inoculated with *L. innocua* at 2 × 10^7^ CFU mounted a significant (*p* < 0.05) IFN-γ+ central memory cell response (see electronic supplementary material, figure S4). Concurrent with the literature, and due to the nature of central memory cells localizing mainly in lymphoid organs, such as the spleen, and effector memory cells localizing in peripheral tissues, we did not observe a robust effector memory cell population responsible for contribution of IFN-γ (see electronic supplementary material, figure S4). Overall, central memory CD8+ T cells provided the largest proportion of IFN-γ produced in mouse spleens after primary infection with Lm.

**Figure 2.**
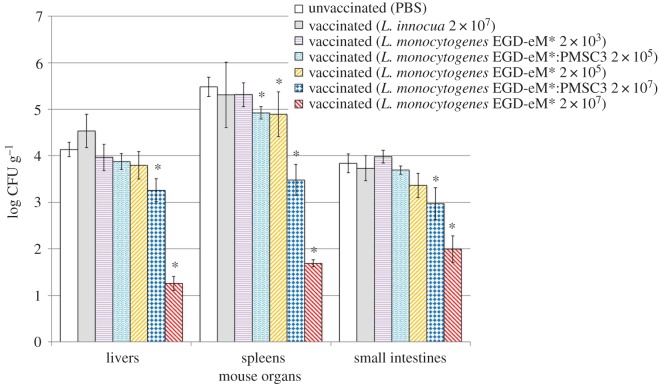
Lm populations recovered from internal organs of mice in the vaccine challenge experiments to evaluate the protective immunity induced by prior exposure to *Listeria*. Female BALB/c 8–10-week-old mice received primary challenge as either (i) oral delivery of the carrier solution (unvaccinated) or (ii) oral inoculation (vaccinated) with a dose corresponding to relevant doses (2 × 10^3^, 2 × 10^5^ or 2 × 10^7^ CFU) in food exposures of *Listeria innocua* (non-pathogenic control), an fV *L. monocytogenes* strain EGD-eM* or *L. monocytogenes* strain EGD-eM*:PMSC3 carrying a virulence-attenuating SNP in *inlA*. Animals were maintained for greater than 40 days after the primary challenge and subsequently orally re-challenged by a high dose (2 × 10^7^ CFU) of fV EGD-eM*; they were euthanized 72 h after this secondary challenge. Organs evaluated are on the *x*-axis. Groups of at least six BALB/c mice were infected at the specified doses and recovery of strains from internal organs was used to define protective immunity to secondary challenge. Organ homogenates were serially diluted and plated on brain–heart infusion broth for bacterial enumeration. Columns represent the mean log_10_ CFU g^−1^ of *L. monocytogenes* strains recovered from internal organs and error bars represent the standard error of the mean. Statistical analyses were performed using a one-way ANOVA using the Tukey test to account for multiple comparisons; asterisks indicate significant differences at the *p* < 0.05 level compared the unvaccinated group. (Online version in colour.)

Overall, the data from the vaccination challenge experiments demonstrated that both the inoculating dose and the strain used for primary infection had a protective effect against subsequent challenge > 40 days later (figure 2). Specifically, compared to unvaccinated animals, no significant protection (*p* > 0.05) was provided by a primary inoculation at 2 × 10^3^ CFU for any organs analysed, when inoculated with the fV strain EGD-eM* (figure 2). Recovery of Lm from the spleens was considered most indicative of systemic Lm infection [5,16]. Notably, significant protection (*p* < 0.05) was provided by both strains EGD-eM* and EGD-eM*:PMSC3 at a primary inoculation of 2 × 10^5^ and 2 × 10^7^ CFU in the spleens of mice when compared with the unvaccinated group (figure 2), with a stronger level of protection elicited with the high dose. Thus, oral vaccination with vA murinized Lm at a level with which this strain is commonly observed in foods (i.e. 2 × 10^5^ CFU) provided small, though statistically significant, protection against subsequent fV Lm challenge in mice. In the small intestines and liver, however, EGD-eM* and EGD-eM*:PMSC3 administered at 2 × 10^5^ CFU did not provide significant protection (*p* > 0.05), protection was only provided when administered inoculum at 1 × 10^7^ CFU for these organs (figure 2). The PCR-RFLP assay revealed only the fV challenge strain was enumerated from organs, as the primary inoculating vA strain was never detected.

### 4.2. Mathematical modelling results

We developed a compartmental mathematical model of immune priming in the human population through natural Lm foodborne exposure. The internal and external validation of the model supported the validity of the model. Comparisons of the model predictions for the US population under the baseline values of model parameters (table 4) and for each of the two sets of values for unidentifiable parameters ($\kappa_{\mathrm{SC}}$, $\kappa_{\mathrm{PC}}$ and *γ*; in table 5) estimated through model calibration are shown in table 6. Both calibration parameter sets produced model predictions that were in good agreement to the known epidemiological data. Applying the model to the Canadian population, the ‘Wide’ calibration parameter set predicts a mean of 228 cases, whereas the ‘Narrow’ set predicts a mean of 194 cases, both of which fit within the previously reported interval of 134–312 for the annual number of listeriosis cases [24]. For the EU, the ‘Wide’ calibration set predicted a mean of 4855 cases and the ‘Narrow’ set predicted a mean of 3768 annual cases, which is similar to the estimated 3730 yearly cases in 2014 adjusted for underreporting [25,26]. At the EU member country level, model predictions based on either set of calibration parameters were not significantly different from the reported cases based on the Wilcoxon signed-rank test (‘Wide’ set: *p* = 0.074, ‘Narrow’ set: *p*-value = 0.7966). Results of sensitivity analysis are shown in electronic supplementary material, figure S6. Briefly, regardless of whether the ‘Wide’ or ‘Narrow’ parameter set is chosen, the model is most sensitive to $\kappa_{V}$, the probability of developing illness when exposed to the fV strain.

**Table 6.** Model predictions of the current number of listeriosis cases and deaths, and per cent colonized annually in the US population under each of the two calibration criteria defined in table 5, compared to available estimates from the literature.

	model predictions		estimates based on literature	source
calibration criterion^a^	Wide	Narrow	not applicable	not applicable
total number of listeriosis cases annually	1929	1638	1591 (557–3161)^b^	[3]
number of cases from virulence-attenuated strains annually (% of total cases annually)	85 (4.3%)	71 (4.3%)	82 (5.1%)	[9]
number of cases from fully virulent strains annually (% of total cases annually)	1846 (95.7%)	1567 (95.7%)	1518 (94.9%)	[9]
number of listeriosis deaths (case fatality) due to any strain annually (% of total cases annually)	265 (13.7%)	225 (13.7%)	256 (16%)	[3]
number of colonized in the US population (% colonized in the US population)^c^	2.34 × 10^7^ (7.8%)	3.3 × 10^7^ (11.01%)	2.4 × 10^5^–2.7 × 10^7^ (0.8–9%)	[19,20]

^a^Parameter values for Wide: *κ*_SC_ = 0.0989, *κ*_PC_ = 0.0992 and *γ* = 0.0359; and Narrow: *κ*_SC_ = 0.144, *κ*_PC_ = 0.146, and *γ* = 0.0268; other parameters as in table 4.

^b^Mean (90% credible interval).

^c^Lm faecal shedding in healthy individuals was represented as colonized compartments in the model in figure 1.

**Figure 3.**
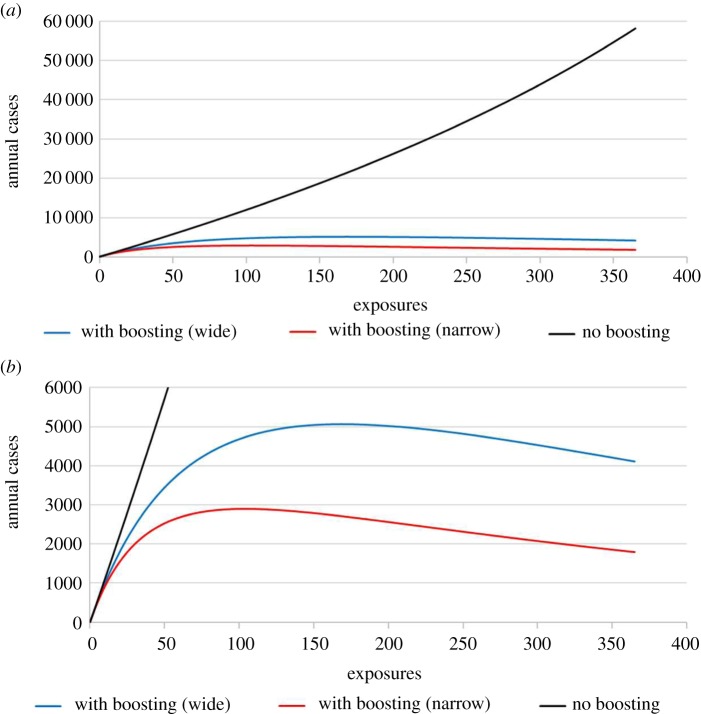
The model predicted the annual number of human listeriosis cases for different assumed frequencies of exposures to *L. monocytogenes* contaminated foods annually (assuming probabilities of exposure to fV and vA strains of 55% and 45%, respectively). Two scenarios are evaluated: (i) immune boosting present with model parameters calibrated using the Wide or Narrow calibration criterion defined in table 5 (shown as ‘with boosting (wide)’ and ‘with boosting (narrow)’) and (ii) no immune boosting present (shown as ‘no boosting’). (*a*) The annual number of cases in a model with no immune boosting compared to predictions in a model with boosting being present. With no immune boosting present, the number of cases continues to grow rapidly and nearly linearly as the annual number of exposures increases while there is a nonlinear relationship between exposures and listeriosis cases with immune boosting present. (*b*) The same prediction as in (*a*) but with a narrower *y*-axis that better matches the epidemiology of listeriosis. With immune boosting present, the number of cases reaches a maximum and then decreases, as exposures are increased. All predictions are from the model at the stable steady state. (Online version in colour.)

The predictions from the model indicate a nonlinear relationship between human Lm foodborne exposures and listeriosis (figure 3). To evaluate implications of immune boosting in this model, we also obtained predictions from a simplified ‘No boosting’ model where individuals could only move from *S* → *I* → *P* → *S*. In comparison to the model with immune boosting, in the ‘No boosting’ model, the predicted number of listeriosis cases continues to grow rapidly and, presumably unrealistically, as the exposure frequency is increased (figure 3). The highest reported incidence of Lm was in 1990, with 0.8 cases per 100 000 people (or approx. 2000 cases) and although contaminations in deli meats, once considered the primary source of Lm, have been reduced by ninefold, observed cases have fallen by less than half [6,7]. While deli meats have not been the only source of Lm, under the ‘No boosting’ model, even just an increase to 50 annual exposures (i.e. approx. doubling the exposure frequency) would increase annual incidence to 6000. This result was not sensitive to the length of immunity (i.e. reciprocal of the value of *γ*); using either the upper or lower bound of *γ* that were set in the Monte Carlo simulation, resulted in the same number of predicted cases when annual exposures were changed, the largest possible value for the parameter space.

**Figure 4.**
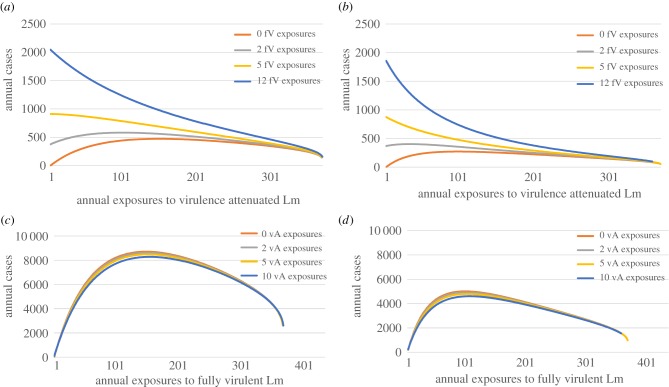
The predicted annual number human listeriosis cases in the model with immune boosting under different scenarios assuming change in the frequency of human foodborne exposure to Lm. In all four panels, the *x*-axis shows different frequencies of exposure to contaminated foods of a single strain (vA in (*a,b*) and fV in (*c,d*)), while the frequency of exposures to the other strain is held constant at one of the four scenarios: 0, 2, 5 or ‘current’ frequency (where ‘current’ means 12 and 10 exposures annually for fV and vA strains, respectively). In (*a,c*), the model predictions are based on parameters from ‘Wide’ calibration, while in (*b*,*d*), they are based on parameters from ‘Narrow’ calibration (as described in table 5). The *y*-axis shows the predicted number of listeriosis cases for each scenario. All predictions are from the model at the stable steady state. (Online version in colour.)

If 50% of all Lm food contaminations are prevented, such that only 11 annual exposures occur per person, the model predicts approximately 1000 cases (1082 under wide parameters and 990 under narrow parameters). As it may be unlikely that both strains are proportionally prevented from entering the food supply, each strain was assessed individually. Controlling only the vA strain resulted in increased listeriosis cases compared to the current level when fV exposures are held at the baseline level (12 exposures per year) or even when fV exposures are reduced by over half to five annual exposures. A 50% reduction in vA, while holding the fV strain the same, resulted in 1987 cases (wide calibration) or 1742 (narrow calibration). Thus, reducing vA alone is predicted to result in a slight increase in the number of listeriosis cases. Controlling only the fV strain resulted in decreased annual cases, regardless of what level the vA strain is held at as long as the frequency of fV exposures is relatively low (at very high frequencies reducing fV may have a negative public health impact) (figure 4). Thus, a 50% reduction in fV, holding vA constant resulted in 1063 cases (wide calibration) or 938 cases (narrow calibration).

## 5. Discussion

For many infectious diseases, the benefit of clinical or subclinical infection is subsequent protective immunity. For infections with temporary immunity, which includes most foodborne infections, evidence suggests that re-exposure to infection may extend the duration of protective immunity, while shortening the length and severity of the infection [27,28]. A previous study demonstrated the level of immunity provided by vaccination with a mutant strain of Lm with reduced virulence appeared very early after vaccination, remained stable for at least four months, then waned slowly afterwards, but was restored and enhanced by a recall exposure [29]. In other words, the immunity is boosted by re-exposure, while re-colonization during the period of immunity usually entails mild or no clinical symptoms [30]. Mathematical modelling of foodborne campylobacteriosis in humans demonstrated that reducing human foodborne exposure to *Campylobacter* may not necessarily lead to a reduction in the occurrence of clinical disease. A mathematical model of Lm in Europe demonstrated a likely rise in annual listeriosis cases as efforts to remove all Lm continue [31], paralleling the observations in several European countries, where contamination levels have fallen over recent decades, yet the reported number of listeriosis cases has increased [8].

In mouse experiments, memory CD8+ T cells generated by vA Lm strains are capable of providing protective immunity when foodborne exposure is at levels that are commonly found in foods.

In this study, we investigated the ability of vA Lm strains carrying a PMSC in *inlA* to (i) elicit a CD8+ T-cell-mediated memory immune response through primary exposure to concentrations commonly found in food and (ii) probed the ability of these strains to provide protective immunity against a subsequent challenge with an fV Lm strain at a high-risk dose. This study herein provided further evidence that after exposure to a vA Lm strain, there is an expansion of *Listeria*-specific CD8+ T effector cells that ultimately lead to a stably maintained pool of central memory CD8+ T cells capable of providing long-term immunity. Overall, the level of immune protection was dependent on strain and dose of initial exposure.

Data from the vaccine challenge experiment demonstrated that protection was not provided by exposure to any *Listeria* strain at a low dose (2 × 10^3^ CFU) and although IFN-γ+ CD8+ T cells were produced at this inoculation level for the fV strain, frequency was lower when compared with strains of either virulence subtype at higher doses (i.e. 2 × 10^5^ and 2 × 10^7^ CFU). This observation suggests that fV Lm strains in the food supply might not play as great a role in priming the immune system to subsequent infection and vA strains are likely to play a larger role in immune priming since they are frequently isolated from foods and found at higher levels of contamination in food compared to fV strains (greater than 10 000-fold higher [32]). Higher inoculum levels pertaining to relevant doses of vA strains per food serving (e.g. 2 × 10^5^ CFU/serving to 2 × 10^7^ CFU/serving) provided protective immunity in both the vaccine challenge model and CD8+ T-cell model. Overall, this study supports that memory CD8+ T cells generated by vA strains are capable of providing protective immunity at levels that are commonly found in foods and that lack of a functional InlA does not impair protection against a secondary Lm infection.

Interestingly, animals inoculated with *L. innocua* at 2 × 10^7^ CFU mounted an IFN-γ+ CD8+ T-cell response similar to animals infected with Lm strains EGD-eM* and EGD-eM*:PMSC3 (see electronic supplementary material, figure S2). Although significantly high levels of IFN-γ+ CD8+ T were observed, *L. innocua* did not provide protection in the vaccine challenge model. A previous study demonstrated primary infection with *L. innocua* resulted in protection against a lethal challenge with the most virulent serotype (4b) of Lm [33]. They found the protective immunity could also be transferred by spleen cells; however, compared with the duration of immunity achieved by primary infection with Lm, the protection induced by infection with *L. innocua* was short-lived and dose-dependent [33]. The induction of protective immunity to high-risk challenge with Lm by a primary infection with *L. innocua* suggests a common immunogenic principle for all *Listeria*, which needs to be further investigated.

The mouse experiments provided promising experimental evidence that single exposure to a murinized vA Lm strain can elicit a CD8+ T-cell response and protection against subsequent challenge with an fV strain. However, humans are exposed to Lm-contaminated foods repeatedly. Therefore, it is important to conduct follow-up experiments to evaluate how the timing and frequency of repeated exposures to Lm may interact with the strain and dose of exposure in terms of the achieved level of elicited immune response and protection against a subsequent challenge with an fV strain. Indeed, one may hypothesize that an additive effect of multiple repeated exposures may induce a stronger level of protection than that elicited with a single exposure to a strain–dose combination tested here, which stresses even more the need for such follow-up experiments.

Given immune boosting, reducing the frequency of exposures to an fV Lm strain would reduce the incidence of listeriosis when the frequency of exposures is already low, whereas reducing exposure to vA strains could have adverse public health impacts.

We developed a novel two-strain mathematical model of human listeriosis with immune boosting and validated it internally in the USA and externally in Canada and the EU. With the model, we are able to understand the implications of removing fV or vA Lm strains from food and to evaluate the possible role of immune boosting. While the purpose of this model is not to suggest new food safety policies, it can provide insight into why current policies may have less than ideal public health outcomes. Given the higher bacterial contamination levels with vA strains, it may be plausible that these contaminations are preferentially found and removed. At the population level, if this is in fact what is occurring, this may not be a beneficial use of resources, as it may be leading to diminished population-level immunity against more virulent strains and an increased incidence of listeriosis.

Removing vA strains completely, without adequately targeting fV strains, is the hypothetical worst-case scenario (figure 4), especially in comparison to proportional removal of both fV and vA strains (figure 3). However, there appears to be room in which both strains are targeted, yet the annual number of cases will remain approximately the same. Maintaining the annual number of cases is obviously more favourable than annual increases, but nonetheless does not move towards improving public health metrics. Thus, there is a need for novel strategies to continue to decrease the annual incidence of listeriosis, including reaching the Healthy People 2020 goal of 0.2 cases per 100 000 people [34]. Although vA strains have a substantially smaller risk of causing human illness compared to fV strains, risk does exist, particularly in extremely immune-compromised individuals. Because natural vaccination via exposure to a vA strain in food is idealistic, the need for a new, intentional intervention strategy, such as a safe vaccine against Lm, may be critically important, e.g. during large-scale outbreaks in naive populations [35,36].

As was expected based on the model set-up, for any given exposure in the model, the probability of becoming colonized is higher than the probability of becoming clinically ill, which corresponds to population-level observations. With approximately 1600 listeriosis cases per year, this is only 0.0005% of the US population, which is over a thousand times lower than the 0.8% of Lm faecal carriage observed in healthy volunteers [19] and even lower than the reported 9% upper range of healthy carriers [20]. Previous work identified reactive T-lymphocytes against Lm in healthy individuals, indicating that it is common to mount an immune response to Lm without being clinically affected [37]. These observations support the existence of colonized individuals as considered in the developed mathematical model and an important factor for considering in future Lm policy.

While this model provides a novel approach to investigating listeriosis, there are nonetheless limitations. Three of the model parameters were uncertain and non-identifiable, which limits our ability to understand their effect on the epidemiology of listeriosis. The parameters describing probability of infection and colonization were scaled to describe the general US population and the model was then accordingly applied and evaluated for the entire US population, which is assumed to be homogeneous. While the elderly, immune-compromised, pregnant and very young are generally the highest risk groups, exposure in the model is nonetheless across all individuals in the USA, including healthy individuals. Because of the absence of data, the model did not assess varying immune response abilities across each of these groups in regard to mounting a protective response. Pregnant women are considered a highly susceptible population but while women themselves generally experience mild illness, the more devastating consequences are for the developing fetus. For pregnant women, previous Lm exposure appears to have no benefit on fetal protection [38]. While the use of an agent-based model could be applied to understand Lm epidemiology, parametrization of such a model would prove challenging and be based largely on assumptions, given the lack of data in regard to strain-level data, including across different groups of people. Additionally, other factors not evaluated in this study may play an important role in the epidemiology and food attribution of listeriosis. Other hypothesized factors that could explain the disparity in foodborne exposure and disease incidence include: (i) pathogen numbers being so low in foods that further reductions are of no clinical benefit, (ii) increased ability to colonize the human intestinal tract, either via evolution of bacterial virulence traits or a change in the human gastrointestinal flora, (iii) shift in bacterial fitness for niche survival; some pathogens may be evolving to more efficiently infect the host, and (iv) a larger population of elderly and immune-compromised individuals at risk. The role of these other factors in the epidemiology of human listeriosis should be considered in parallel to the hypothesized role of immune boosting evaluated here before designing new public health strategies.

Quantitative estimates from mice experiments could not be used directly in the model of listeriosis in a human population due to obvious differences between mice and men. This is a challenge common to many human infectious diseases where experimentation would be unethical and animal models are used instead. However, findings from the mouse experiments effectively informed the structure of the mathematical model of human listeriosis. In that sense, mathematical modelling allowed novel integration of the within-host and population-level scales to improve understanding of the effect of human foodborne exposures to Lm strains of different virulence on the public health burden of listeriosis. The model can be further improved as more experimental data become available, including on whether the frequency and timing of repeated exposures to Lm may have an effect on the disease incidence.

## 6. Conclusion

While findings from this study should not be misinterpreted as a recommendation for food safety professionals to reduce efforts in continuing to control Lm, our experimental findings and modelling predictions support that exposure to vA strains is protective against subsequent exposure to fV strains at the individual level in a mouse model and the human population level. In addition to further preventing Lm from entering the food supply, new efforts could focus on development of vaccines against Lm for at-risk populations.

## Supplementary Material

Supplemental materials

## Data accessibility

The mouse experimental data, the code for the model and Monte Carlo simulation data can be accessed at https://github.com/IvanekLab/Lmcompartmentalmodel.
