# Supplementary material for: Public health impact of foodborne exposure to naturally occurring virulence-attenuated Listeria monocytogenes: inference from mouse and mathematical models
Source: Interface Focus. 2019 Dec 13;10(1):20190046. doi: 10.1098/rsfs.2019.0046 (PMC6936009; doi:10.1098/rsfs.2019.0046)
Supplement: Supplemental materials [file rsfs20190046supp1.docx]

***Supplementary material***

**Methods and materials**

**Experimental mouse model**

*Genetic engineering of the virulence-attenuated (vA) murinized Listeria monocytogenes (Lm) strain carrying an InlA PMSC***.** The native form of E-cadherin found in mice and rats differs from the human form of E-cadherin by a single amino acid; this substitution prohibits InlA from binding to E-cadherin in mice and rats (Lecuit et al., 1999). The guinea pig and gerbil isoform of E-cadherin however, interacts with InlA (Lecuit et al., 2001), but are limiting for immunological studies due to lack of reagents, which are commonly developed for mice. To compensate for the lack of specificity between Lm and the murine isoform of E-cadherin, a previous study ‘murinized’ Lm (EGDe-M*; Monk et al., 2010). More specifically, Monk et al. (2010) re-created the murinized Lm strain initially designed by Wollert and colleagues (Wollert et al., 2007) using a technique that minimized the likelihood of secondary mutations and incorporated *Listeria*-optimized codons encoding the altered amino acids. Herein, we introduced the most common InlA PMSC mutation (PMSC type 3) into EGD-eM* to enable characterization of vA Lm strains in an oral mouse infection model. This mutant was constructed using the allelic exchange mutagenesis procedure, as previously discussed (Camili et al., 1993), with the suicide shuttle vector pKSV7 that carries a temperature-sensitive *ori*R for *Listeria*. Primer sequences for mutant constructs are listed in Table S1. This procedure generated a mutant carrying PMSC type 3 by integrating a stop codon (TAG) for amino acid Tyrosine (TAC) at position 700 by a single nucleotide substitution of a G for a C at nucleotide position 2100; this approach, therefore, yielded a strain that differed from its parent strain by the presence of a SNP responsible for the vA PMSC type 3 in InlA. The mutant construct was verified by sequencing the 3’ region of *inlA* as detailed previously (Table S1; Nightingale et al., 2005).

*Bacterial growth conditions for cell culture invasion assays and oral inoculum preparation.* Bacterial cultures for invasion assays were grown in brain heart infusion (BHI; Becton Dickson, Sparks, MD) broth at 30°C statically for 18h as described previously (Nightingale et al., 2005). Bacterial cultures used for mouse infection experiments were grown at 37°C with shaking overnight for 12-18 hours. Bacterial counts for cell invasion assays and mouse inocula were determined by plating appropriate serial dilutions on BHI agar in parallel with each experiment. Inoculum for oral mouse infection was prepared as described in Monk et al. (2010); overnight cultures were centrifuged (7,000 x g for 5 min), serially diluted in Phosphate Buffered Saline (PBS) to the desired CFU, and resuspended in PBS containing 100 mg/ml of calcium carbonate (CaCO_3_) in a total volume of 100μl.

*Bacterial growth curves.*  The resultant vA murinized strain (EDG-eM*:PMSC3) and the fully-virulent (fV) EGD-eM* strain were evaluated for growth defects using a standard growth curve assay where growth characteristics of the mutants were compared to the parent strain, as previously described (Oliver et al., 2010). Briefly, BHI broth was inoculated with populations of the Lm strains (6.68 log_10_ CFU/ml) and was maintained at 37°C for 12h with agitation. OD was read every hr for 12 hrs and bacteria were plated at time point 0 hr, OD=.4, 8 hr, 10 hr, and 12 hr. Three biological replicates of each assay were performed.

*Cell culture invasion assays***.** The resultant vA murinized strain (EDG-eM*:PMSC3) and the fV EGD-eM* strain were evaluated for cell invasion efficiencies using cell culture invasion assay where invasion capabilities of the mutants were compared to the parent strain. Assays were performed in both human intestinal epithelial Caco-2 cell (American Type Culture Collection) and mouse intestinal epithelial CT-26 cell lines. Invasion assays were performed essentially as previously described (Nightingale et al., 2005). Briefly, semiconfluent monolayers were inoculated with approximately 2x10^7^ Lm cells/well (each strain was assayed in duplicate wells), and the exact inoculum was determined by plating on BHI agar. Cell monolayers inoculated with Lm were incubated at 37°C for 30 min, followed by three washes with PBS to remove nonadherent bacteria and then the addition of fresh medium without antibiotics. Medium containing 150 μg/ml gentamicin was added 45 min postinoculation to kill extracellular bacteria. At 90 min postinoculation, infected cell monolayers were washed three times with PBS and subsequently lysed by addition of cold sterile deionized water with vigorous pipetting to release intracellular bacteria. Intracellular Lm cells were enumerated by spread-plating appropriate dilutions of the lysed cell suspensions on BHI agar. An uninoculated BHI broth was included as a negative control in each invasion assay. Three biologically independent assays for each strain were performed in both cell lines. Invasion efficiency was reported as the natural log of the percentage of initial inoculum recovered after enumeration of intracellular bacteria.

*Statistical analysis of cell culture invasion assays***.** Natural log-transformed Caco-2 cell and CT-26 cell invasion efficiency data (intracellular populations were expressed as a percentage of the inocula) for Lm strains were analyzed using a one way analysis of variance (ANOVA) with isolate as a class variable as implemented in the MIXED procedure of SAS (Statistical Analysis Systems Software, Cary, NC). Mean separation on independent variables was achieved using Tukey’s honest significant difference (HSD) test for multiple comparisons. Natural log transformations were used to satisfy the assumptions of the ANOVA; a Shapiro-wilk’s test for normality and Levene’s test for homogeneity were run to ensure data met assumptions of the ANOVA. Probabilities of <0.05 were considered statistically significant.

*Oral mouse infection***.** For *in vivo* virulence characterization of the mutant constructs used in this study, animals were inoculated at 2x10^9^ CFU and maintained for 72 hours post-inoculation before euthanization via CO_2_ asphyxiation. Other experiments are described in the main body of the article.

*Organ processing for microbiological analysis.* Post-euthanasia, select internal tissues (i.e., liver (gall bladder removed), spleen, mesenteric lymph nodes and small intestine) were aseptically removed, weighed and processed for microbiological analyses to detect and enumerate resultant Lm. Small intestine samples were washed twice with PBS and subsequently treated with gentamicin (150 μg/ml in Dulbecco’s modified Eagle medium (DMEM; Gibco, Life Technologies, Grand Island, NY)) for 90 minutes at room temperature to eliminate extracellular microflora. Small intestines were then washed twice again with PBS prior to homogenization in order to wash off residual antibiotic residues. Organs were homogenized in cold PBS (10ml for livers; 5ml for spleens and small intestines; 3ml for mesenteric lymph nodes) in sterile 50ml conical tubes using a tissue homogenizer probe (Power gen 500; Fisher Scientific). Bacterial numbers in select organs were determined by plating appropriate dilutions on BHI agar and incubating overnight at 37°C. Selective enrichment of organ homogenates was also performed to allow for detection of Lm in samples having low bacterial numbers. More specifically, 1 ml of organ homogenate was selectively enriched into 9 ml of *Listeria* enrichment broth (LEB; Difco, Sparks, MD) at 30°C for 48 h. Aliquots (100μl) of enrichments were streaked onto Oxford plates (Difco; Oxoid, Hampshire, United Kingdom) and plates were incubated at 35°C for 48 h. A previously described PCR-RFLP assay (Nightingale et al., 2005) was used to characterize resultant colonies from each Lm-positive organ for animals vaccinated with EGD-eM*:PMSC3 to differentiate from the challenge strain. Five colonies were screened with the PCR-RFLP assay per positive plate.

*Spenocyte processing and isolation of dentritic cells (DCs) and CD8+ T cells***.** In the experiments to evaluate the CD 8+ T-cell immune response, mice were given a booster immunization of their appropriate strain-dose combination 72 hours prior to euthanization to initiate a recall immune response. Following euthanization, spleens were aseptically removed and gently pressed through a cell strainer (70uM nylon mesh) in Hank’s balanced salt solution (HBSS; Life Technologies, Grand Island, NY) supplemented with 5% low IgG fetal bovine serum (FBS; HyClone, Logan, Utah) to obtain a single-cell suspension of spenocytes. Red blood cells were subsequently lysed via PharmLyse buffer (BD Pharmingen, San Jose, CA) and spenocytes (approx. 1x 10^8^) were subjected to positive selection for DCs via the CD11c MicroBeads mouse kit (MACS Miltenyi Biotec, Auburn, CA), followed by negative selection for CD8+ T cells via the CD8a+T Cell Isolation Kit II for the mouse (MACS Miltenyi Biotec, Auburn, CA).

*Dendritic cell (DC) activation and in vitro T cell-stimulation culture***.** Protective immunity requires infection with Lm that is capable of escaping the vacuole (i.e. strains which express LLO). While in the cytosol, Lm secretes a small number of proteins (Villanueva et al., 1995). CD8 T cells specific for four epitopes from only three different proteins constitute most of the response (Busch et al., 1998). The immunodominant protein, LLO, is the most antigenic of these secreted proteins and thus a large percentage of CD8 T cells are specific for epitopes pertaining to this protein (Haring and Harty, 2006).

For DC activation *ex vivo*, freshly isolated splenic DCs were pulsed with 10ug/ml of the *Listeria* specific peptide LLO^91-99^ (Anaspec, Fremont, CA) in DMEM + 5% FBS for 4 hours (37°C with gentle shaking) and subsequently treated with Mitomycin C (Fisher Bioreagents, Fairlawn, NJ) for 20 min at 37°C to prevent DCs from producing cytokines known to stimulate T cells independently of their T cell receptor (TCR). DCs were then washed 3 times with DMEM + 5% FBS. In a tissue culture plate (Corning Costar, Corning, NY), DCs were cultured with purified populations of CD8+ T cells *ex vivo* at a ratio of 1 DC per 2.5-5 CD8+ T cells overnight in DMEM + 5% FBS for approximately 12 hours in the presence of GolgiPlug (BD, San Jose, CA) to prevent protein secretion.

In the immune boosting experiments to evaluate the recall of the CD8+ T cell immune responses (Table 2), after primary inoculation with different dose-strain combinations, mice were maintained for >40 days before receiving a booster immunization of the same strain-dose combination as during primary inoculation. On day 3 (72 hours) after the immunization booster, splenocytes were harvested and cultured ex vivo with LLO91-99 peptide, then stained for the cell surface markers CD8beta, CD44, and CD62L, subsequently fixed and permeabilized, and stained for intracellular accumulation of IFN-gamma (Van Stelten, 2014, page 95-96).

*Intracellular interferon-γ.* The frequency of IFN-γ secreting CD8+ T lymphocytes specific for Lm was determined for animals >40 days post-infection by cytokine staining using the Cytofix/Cytoperm™ PlusFixation/Permeabilization Kit (BD Pharmingen, San Jose, CA).   Following culture, lymphocytes were stained for the cell surface expression of CD8β (single stain; PE rat anti-mouse CD8b.2 antibody BD clone 53-5.8), and co-stained with CD62L (FITC rat anti-mouse CD62L antibody BD clone MEL-14) and CD44 (PE-Cy 7 rat anti-mouse CD44 antibody BD clone IM7); after which cells were fixed and permeabilized and subsequent intracellular staining of IFN-γ was determined using rat anti-mouse Alexa Fluor 647 INF-γ antibody (BD clone XMG1.2), according to the kit manufacturer’s instructions. Percentage of CD8+ INF-γ+ T cells, along with memory phenotyping via CD44/CD62L expression, was determined by flow cytometry on the Accuri C6 (BD Pharmingen, San Jose, CA) and further analysis was performed through FlowJo (TreeStar, Ashland, OR).

*Statistical analysis of mouse infection experiments***.** For oral mouse infections characterizing the virulence of the mutant constructs, two animals were each infected with one of the three strains per experiment and three biologically independent replications of each experiment were performed. The recovery levels (in log_10_ CFU/g) of Lm from internal organs were used as the main measure of virulence.

For mouse CD8 T cell experiments designed to evaluate the frequency of IFN-γ secreting CD8+ T lymphocytes specific for Lm, a minimum of four animals were infected in each of the seven strain/dose treatment groups. Samples analyzed for each animal included: CD8+ T cells, CD8+ T cells with DCs, and CD8+ T cells with DCs cultures with LLO_91-99_ peptide. All data presented here represents the population of CD8+ T cells cultured with DCs presenting LLO antigen. The percent positivity of IFN-γ from the total CD8+ T cell population, the effector memory population (CD44+, CD62L-), the central memory population (CD44+, CD62L+), and naïve cell population (CD44-, CD62L+), was used as the main measure of response in each strain/dose treatment.

**Mathematical modeling**

*Compartmental model***.** Ordinary Differential Equations for the model depicted in Figure 1 and with parameters defined in Table 4 are given below:

$$\frac{dS}{dt} =B-\left[ \lambda_{A}k_{A}+\lambda_{A}\kappa_{SC}+\lambda_{V}\kappa_{V}+\lambda_{V}\kappa_{SC}+d \right]S+\gamma[P_{A}+P_{V}]$$

$$\frac{dI_{A}}{dt}=\lambda_{A}k_{A}S-[\alpha+\mu+d]I_{A}$$

$$\frac{dC_{A}}{dt}=\lambda_{A}(\kappa_{SC}S+\kappa_{PC}P_{V}+\kappa_{PC}P_{A})-[\alpha+d]C_{A}$$

$$\frac{dP_{A}}{dt}=\alpha[C_{A}+I_{A}]-{(\lambda}_{A}\kappa_{PC}+\lambda_{V}\kappa_{SC}+ \gamma+d)P_{A}$$

$$\frac{dI_{V}}{dt}=\lambda_{V}\kappa_{V}S-[\alpha+\mu+d]I_{V}$$

$$\frac{dC_{V}}{dt}=\lambda_{V}(\kappa_{SC}S+k_{PC}P_{V}+\kappa_{PC}P_{A})-[\alpha+d]C_{V}$$

$$\frac{dP_{V}}{dt}=\alpha[C_{V}+I_{V}]-{(\lambda}_{V}\kappa_{PC}+\lambda_{A}\kappa_{PC}+ \gamma+d)P_{V}$$

*Monte Carlo simulation.* Three values in the model ($\kappa_{SC}$, $\kappa_{PC,}$ and γ) were evaluated using Monte Carlo simulation. Initial ranges for the probabilities $\kappa_{SC}$ and $\kappa_{PC}$ were extrapolated from previous reports of Lm detection in the feces of healthy volunteers ranging from 0.8% (Grif et al., 2001) to 9% (Ralovich, 1984), using trial and error to find a wide range of plausible values for Monte Carlo simulation. Based on that plausible range $\kappa_{SC}$ was simulated as:

$\kappa_{SC} \sim Uniform[0, 0.202]$

To allow for possibilities that $\kappa_{PC}$ may be larger, smaller or equal to the value of $\kappa_{SC}$, the value of $\kappa_{PC}$ was modeled as a function of $\kappa_{SC}$ such that

$\kappa_{PC}=x\kappa_{SC}$

where Factor *x* was assumed to be distributed as:

$x\sim Uniform\left[ 0.5, 1.5 \right]$

Reports on the length of immunity against consumed Lm have ranged from less than a year to up to nearly lifelong (Pamer et al., 2004; Lavi et al., 2008). The rate of waning immunity, γ, was therefore set as,

$\gamma\sim Uniform[6x{10}^{-5}, 0.07]$

where the lower bound corresponds to immunity lasting 45 years and the upper bound corresponds with immunity lasting two weeks, since γ is equal to the reciprocal of the average length of immunity.

**Results**

In this study, site directed mutagenesis was used to introduce InlA PMSC3 into EGD-eM^*^, a previously murinized Lm strain (Monk et al., 2010). Normal growth and viability of the resultant Lm mutant construct (EGD-eM*:PMSC3), along with other Lm strains used in this study, were investigated (EGD-e and EGD-eM^*^) (Table S1). For growth curves, BHI broth was inoculated with populations of the three strains (6.68 log_10_ CFU/ml) and was maintained at 37^o^C for 12h. There were no differences in growth between isolates at 37°C and all strains had exceeded 9.7 log_10_ (CFU/ml) by 12h. For Lm strains EGD-e, EGD-eM^*^, EGD-eM^*^:PMSC3, and *L. innocua* (non-pathogenic control; does not express InlA) cell invasion efficiencies were compared in Caco-2 and CT-26 cell monolayers (Figure S1 A & B).

vA murinized Lm showed reduced invasion efficiency in mouse CT-26 cells, as well as reduced infection capability in the intragastric murine model. Mice infected with EGD-eM^*^ showed significantly higher (*P<0.05*) bacterial levels in the liver, spleen, mesenteric lymph nodes, and small intestines as compared to EGD-e at 72 hours post-infection. EGD-eM^*^:PMSC3 only displayed attenuated virulence, as determined by lower bacterial loads, in livers, spleens, and mesenteric lymph nodes, but not in small intestines (Figure S2) (Van Stelten, 2014, page 113). Significant differences were not observed between EGD-eM^*^ and EGD-eM^*^:PMSC3 in the gut associated lymphoid tissue (GALT), which could be explained by murinization of Lm mediating not only E-cadherin dependent internalization but also N-cadherin dependent internalization leading to enhanced innate immune response and inflammation within these tissues (Tsai et al., 2013).

To address the contribution of effector and central memory CD8+ T cells in mediating long-lasting immunity after primary inoculation with either the fV EGD-eM* or the vA strain EGD-eM*:PMSC3, the expression of the cell surface adhesion molecules CD62L and CD44 were quantified (Figure S4).

| **Table S1.** Description of primer sequences used for allelic exchange mutant constructs | | | | |
| --- | --- | --- | --- | --- |
| Primer name | Primer description, target and direction | | Primer sequence (5’ to 3’) | Reference |
| AVS-Marq147 (A) | SOEing primer for*inlA* PMSC type 3 | Forward | AACTGCAGCTGGGAATTTATT | Nightingale et al., 2005 |
| AVS-Marq148 (C) | SOEing primer for*inlA* PMSC type 3 | Forward | CATTACGCTGTAGGCTCAATT | Nightingale et al., 2005 |
| AVS-Marq149 (B) | SOEing primer for*inlA* PMSC type 3 | Reverse | GTAAATTGAGCCTACAGCGTA | Nightingale et al., 2005 |
| AVS-Marq150 (D) | SOEing primer for*inlA* PMSC type 3 | Reverse | GCGGTACCTTGCTTGATTGGC | Nightingale et al., 2005 |
| AVS42 XF_PMSC3 | Sequencing primer for verification of PMSC type 3 | Forward | GTGACGCAGCCACTTAAGGC | Nightingale et al., 2005 |
| AVS43 XR_PMSC3 | Sequencing primer for verification of PMSC type 3 | Reverse | GTGTCACTGCATCTGTCACAC | Nightingale et al., 2005 |
| AVS44 XIF_PMSC3 | Internal sequencing primer for verification of PMSC type 3 | Forward | AATTTACGAAAAATCCTGTGG | Nightingale et al., 2005 |
| AVS45 XIR_PMSC3 | Internal sequencing primer for verification of PMSC type 3 | Reverse | GAAGTCCTTCATAGTCTACTG | Nightingale et al., 2005 |

**Table S2.** As part of external validation of the model in Figure 1, we compared the model predicted and reported annual number of listeriosis cases in European Union member countries in 2014. The model was evaluated with 38 annual exposures per person (Ricci et al, 2017) and under the same assumption that 45% of contaminations were with the virulence-attenuated strains of *L. monocytogenes* as it was assumed in the model run for the US population. The confirmed cases were previously reported by the European Center for Disease Control and Prevention (2015) and were adjusted by a factor of 1.7 to account for underreporting (Ricci et al, 2017). The countries of Liechenstain, Portugal and Cyprus were excluded, as no cases were reported or no information was provided. The population of each country in 2014 was obtained from Eurostat (Eurostat, 2019). “Wide” and “Narrow” parameter values correspond to the calibration criteria described in Table 5.

| Country | Population | Reported cases | Reported cases adjusted for underreporting | Predicted cases (based on “Wide” parameter values) | Predicted cases (based on “Narrow” parameter values) |
| --- | --- | --- | --- | --- | --- |
| Austria | 8.5 million | 49 | 83.3 | 82.53 | 64.06 |
| Belgium | 11.18 million | 84 | 142.8 | 108.55 | 84.26 |
| Bulgaria | 7.246 million | 10 | 17 | 70.35 | 54.61 |
| Croatia | 4.247 million | 4 | 6.8 | 41.24 | 32.01 |
| Czech Republic | 10.51 million | 38 | 64.6 | 102.05 | 79.21 |
| Denmark | 5.627 million | 92 | 156.4 | 54.63 | 42.41 |
| Estonia | 1.316 million | 1 | 1.7 | 12.78 | 9.92 |
| Finland | 5.451 million | 65 | 110.5 | 52.93 | 41.08 |
| France | 66.13 million | 374 | 635.8 | 642.08 | 498.4 |
| Germany | 80.77 million | 597 | 1014.9 | 784.23 | 608.74 |
| Greece | 10.93 million | 10 | 17 | 106.12 | 82.38 |
| Hungary | 9.877 million | 39 | 66.3 | 95.90 | 74.44 |
| Iceland | 325671 | 4 | 6.8 | 3.16 | 2.45 |
| Ireland | 4.638 million | 15 | 25.5 | 45.03 | 34.96 |
| Italy* | 60.78 million | 52 | 88.4 | 590.14 | 458.08 |
| Latvia | 2.001 million | 3 | 5.1 | 19.43 | 15.08 |
| Lithuania | 2.943 million | 7 | 11.9 | 28.57 | 22.18 |
| Luxembourg | 549680 | 5 | 8.5 | 5.34 | 4.14 |
| Malta | 429424 | 1 | 1.7 | 4.17 | 3.24 |
| Netherlands | 16.83 million | 90 | 153 | 163.41 | 126.84 |
| Norway | 5.108 million | 29 | 49.3 | 49.60 | 38.5 |
| Poland | 38.02 million | 86 | 146.2 | 369.15 | 286.55 |
| Romania | 19.95 million | 5 | 8.5 | 193.70 | 150.36 |
| Slovakia | 5.416 million | 29 | 49.3 | 52.59 | 40.82 |
| Slovenia | 2.061 million | 18 | 30.6 | 20.01 | 15.53 |
| Spain | 46.51 million | 161 | 273.7 | 451.58 | 350.53 |
| Sweden | 9.645 million | 125 | 212.5 | 93.65 | 72.69 |
| United Kingdom | 64.35 million | 201 | 341.7 | 624.80 | 484.99 |


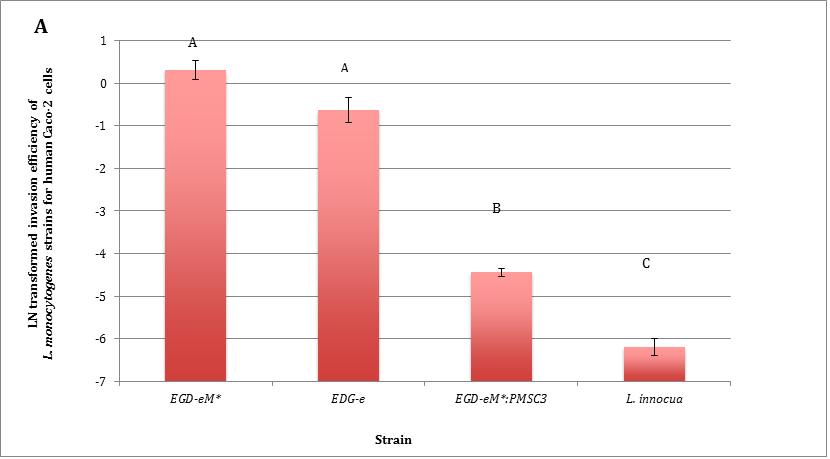

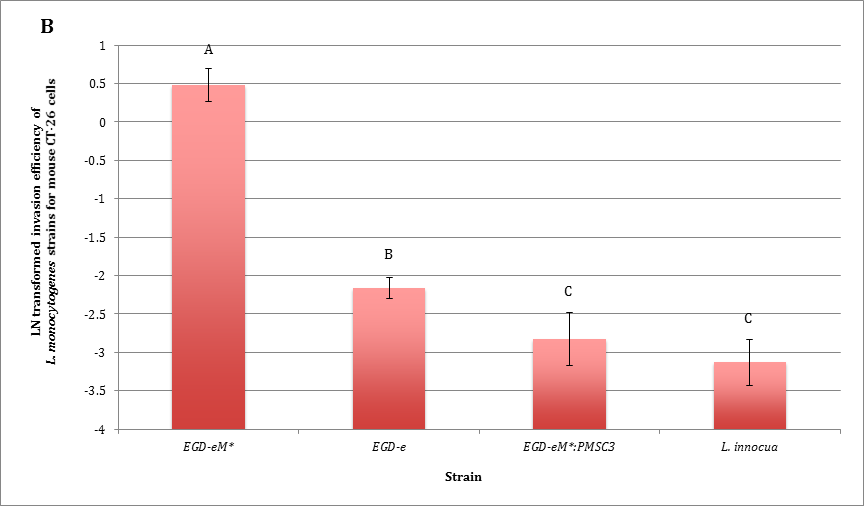


**Figure S1.** Natural log (LN)-transformed human Caco-2 (A) and mouse CT-26 (B) cell invasion efficiencies for a non-murinized *. monocytogenes* strain and murinized *L. monocytogenes* strains with and without a virulence-attenuating SNP in *inlA*.  Strains used are indicated on the *x* axis and are as follows: EGD-e is a laboratory control strain representing wildtype *L. monocytogenes*, EGD-eM* is a fV murinized *L. monocytogenes*strain (Monk et al., 2010), EGD-eM*:PMSC3 is a murinized strain where InlA PMSC3 was introduced via site directed mutagenesis into the EGD-eM* background, and *L. innocua* was used as a non-pathogenic control.  The natural log-transformed invasion efficiency of each strain is represented on the *y* axis.  All strains were assayed in duplicate in each independent experiment, and three biologically independent experiments of the invasion assays were performed.  Columns represent mean invasion efficiency and error bars represent the standard error of the mean.  Statistical analyses were performed using a one-way analysis of variance (ANOVA) using Tukey’s HSD to account for multiple comparisons; differing letters indicate significantly different invasion efficiencies at the *P*<0.05 level.


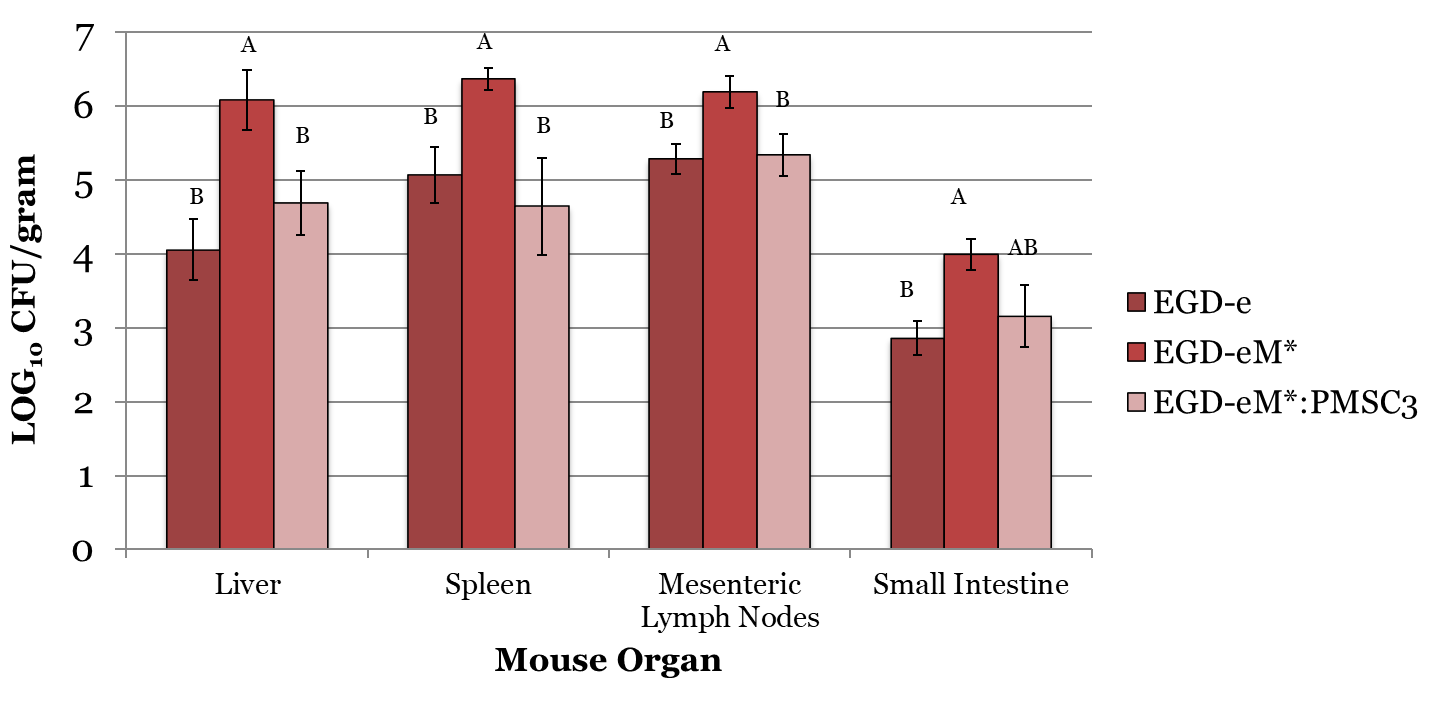


**Figure S2.**  *L. monocytogenes* populations recovered from 8-10 week old female BALB/c mice orally infected with non-murinized and murinized *Listeria* strains with and without a virulence-attenuating SNP in *inlA* at 72 hours post-infection.  Strains evaluated are on the *x* axis and are as follows: EGD-e is a laboratory control strain representing wildtype *L. monocytogenes*, EGD-eM* is a fV murinized *L. monocytogenes*strain (Monk et al., 2010), EGD-eM*:PMSC3 is a murinized strain where InlA PMSC3 was introduced via site directed mutagenesis into the EGD-eM* background. Mice (n=6) were infected at 2x10^9^ CFU and recovery of strains from internal organs was used to define infection. Three biologically separate replicates including 2 mice per strain were performed.  Organ homogenates were serially diluted and plated on BHI for bacterial enumeration. Columns represent mean log_10_ CFU/gram of *L. monocytogenes* strains recovered from internal organs and error bars represent the standard error of the mean. Statistical analysis were performed using a one-way analysis of variance (ANOVA) using Tukey’s HSD to account for multiple comparisons; differing letters indicate significant differences at the *P*<0.05 level.


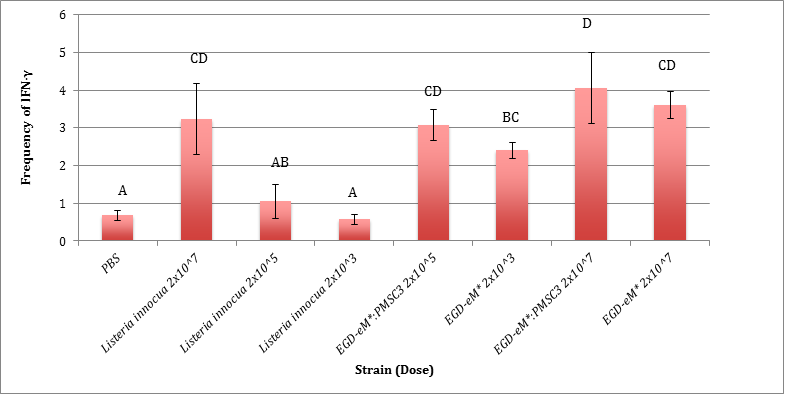


**Figure S3.** Frequency of total IFN-γ from CD8+ T cell populations recovered from spleens of 8-10 week old female BALB/c mice orally infected with murinized *L. monocytogenes* strains with and without a virulence-attenuating SNP in *inlA*, *L. innocua* and PBS (negative control) >40 days after primary inoculation with the same strain-dose combination.  Strain/dose treatment combinations evaluated are on the *x* axis. The *y*-axis represents % of total CD8+ T cells producing IFN-γ. A minimum of 4 mice were inoculated with each strain/dose treatment combination. Columns represent mean % IFN-γ and error bars represent the standard error of the mean.  Statistical analysis were performed using a one-way analysis of variance (ANOVA) using LSD to account for multiple comparisons; differing letters indicate significant differences at the *P*<0.05 level.


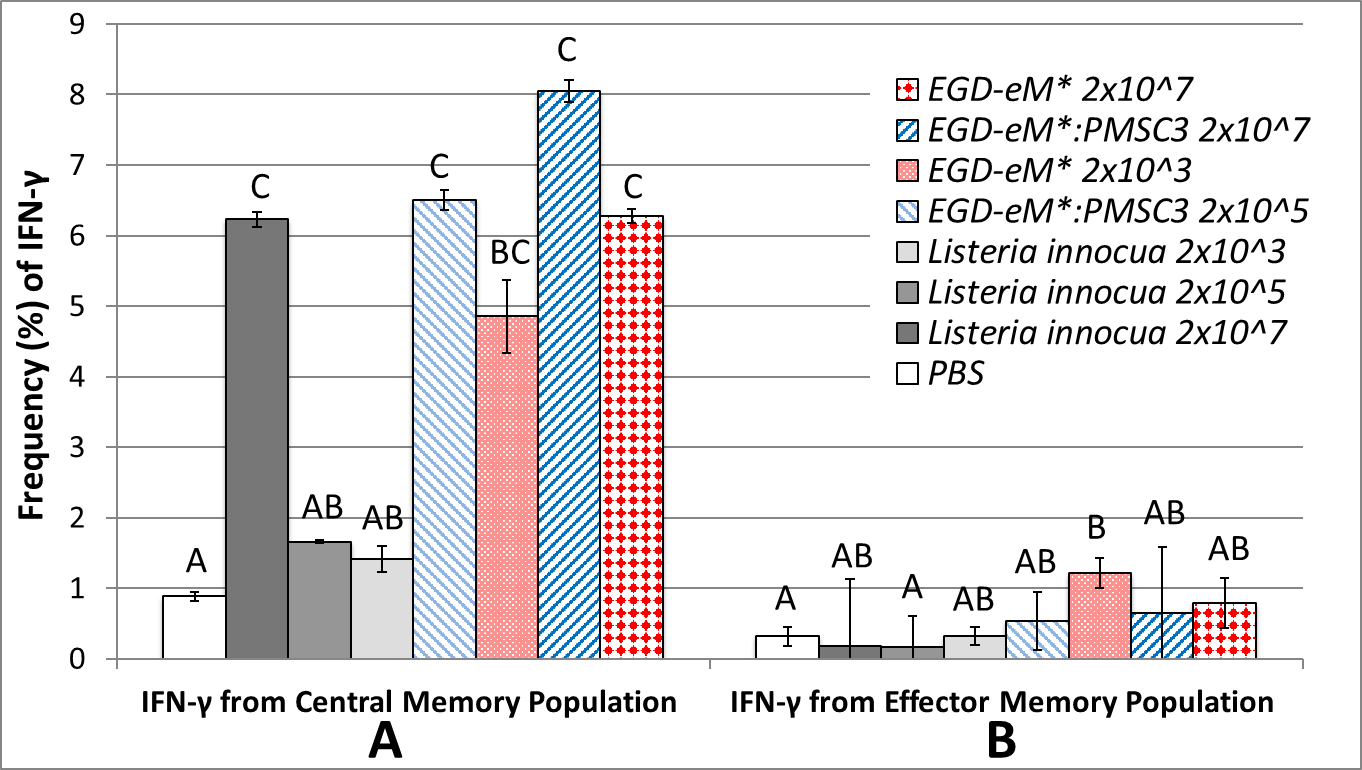


**Figure S4.** Frequency of IFN-γ from CD8+ T cell populations for A) central memory populations (CD62L+ CD44+) and B) effector memory populations (CD62L- CD44+) recovered from spleens of 8-10 week old female BALB/c mice orally infected with murinized *L. monocytogenes* strains with and without a virulence-attenuating SNP in *inlA*, *L. innocua* and PBS (negative control) >40 days after primary inoculation with the same strain-dose combination.  Strain-dose treatment combinations evaluated are on the *x* axis. The *y* axis represents % of total CD8+ T cells producing IFN-γ. A minimum of 4 mice were inoculated with each strain/dose treatment combination. Columns represent mean % IFN-γ  and error bars represent the standard error of the mean.  Statistical analyses were performed using a one-way analysis of variance (ANOVA) using LSD to account for multiple comparisons; differing letters indicate significant differences at the *P*<0.05 level.


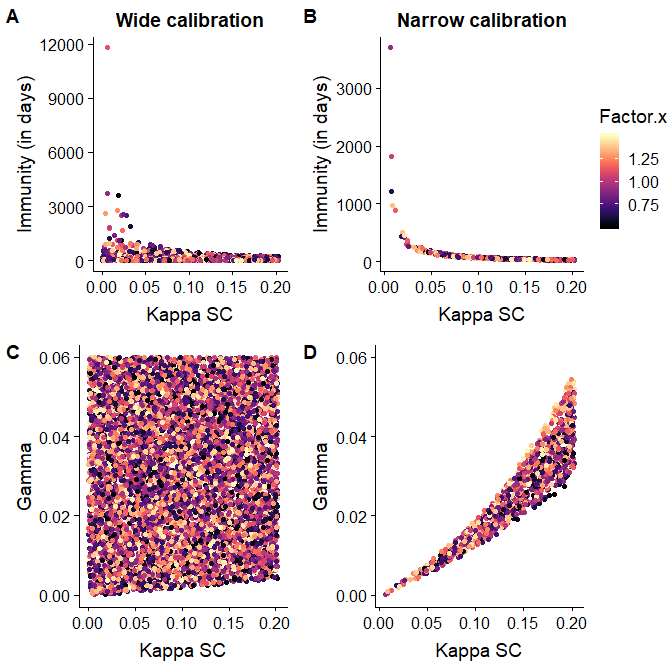


**Figure S5.** Combinations of values of non-identifiable model parameters (κ_SC_, κ_PC_ and γ, defined and parameterized in Tables 4 and 5) obtained by selecting iterations from Monte Carlo simulations where the predicted annual number of listeriosis cases matched one of the two used calibration criteria. The x-axis gives the value for  $\kappa_{SC}$or, the probability of an individual in the Susceptible compartment becoming colonized after an exposure.  In panels A and B, the y-axis corresponds to the length of immunity (in days) obtained as reciprocal of the parameter gamma. In panels C and D, the y-axis gives the parameter gamma, or the rate of immunity loss, corresponding to the length of immunity in panels A and B, respectively.  The color of each point denotes the Factor *x* that $\kappa_{SC}$is multiplied by to yield $\kappa_{PC}$ (the Factor *x* was allowed to vary between 0.5 to 1.5 to allow possibilities of $\kappa_{PC}$ being smaller, equal or larger than $\kappa_{SC}$). In panels a. and c. the “Wide” calibration criterion was used meaning that iterations with the predicted listeriosis cases within the range of 557-3161 were selected for estimation of the non-identifiable parameters. In panels b. and d., the “Narrow” calibration criterion was used so that we selected iterations where the predicted listeriosis cases were within the range of 1500-1700.

A

B

**Figure S6.** The sensitivity of the model to individual parameters was evaluated by scaling each parameter by 1.5 and 0.5 and individually assessing the effects on the predicted annual number of listerisis cases when all other parameters were held constant. Panel A shows results when using the “Wide” combination of values of non-identifiable parameters. Panel B shows the results when the “Narrow” combination of values of non-identifiable parameters was used. Overall, in both scenarios the predictions are most sensitive to $\kappa_{V},$ or the probability of illness given an exposure to the fV strain.

**References** (specific to Supplementary material)

1. **Busch DH, Pilip IM, Vijh S, Pamer EG.** 1998. Coordinate regulation of complex T cell populations responding to bacterial infection. Immunity. **8**:353e362.
2. **Camilli A, Tilney LG, Portnoy DA.** 1993. Dual roles of plcA in *Listeria monocytogenes* pathogenesis. Molecular microbiology. **8:**143-157.
3. **Eurostat**. 2019. Population on January 1 by age and sex. Retrieved from <http://appsso.eurostat.ec.europa.eu/nui/show.do?dataset=demo_pjan&lang=en>
4. **Haring JS, Harty JT.** 2006. Aberrant contraction of antigen-specific CD4 T cells after infection in the absence of gamma interferon or its receptor. Infect. Immun. **74**:6252-63.
5. **Lecuit M, Dramsi S, Gottardi C, Fedor-Chaiken M, Gumbiner B, Cossart P.** 1999. A single amino acid in E-cadherin responsible for host specificity towards the human pathogen *Listeria monocytogenes*. The EMBO journal. **18:**3956-3963.
6. **Lecuit M, Vandormael-Pournin S, Lefort J, Huerre M, Gounon P, Dupuy C, Babinet C, Cossart P.** 2001. A transgenic model for listeriosis: role of internalin in crossing the intestinal barrier. Science. **292:**1722-5.
7. **Monk IR, Casey PG, Hill C, Gahan CG**. 2010. Directed evolution and targeted mutagenesis to murinize *Listeria monocytogenes* internalin A for enhanced infectivity in the murine oral infection model. BMC Microbiol. **10:**318.
8. **Nightingale KK, Windham K, Martin KE, Yeung M, Wiedmann M.** 2005. Select *Listeria monocytogenes* subtypes commonly found in foods carry distinct nonsense mutations in *inlA*, leading to expression of truncated and secreted internalin A, and are associated with a reduced invasion phenotype for human intestinal epithelial cells. Appl. Environ. Microbiol. **71:**8764-8772.
9. **Pamer EG.** 2004. Immune responses to Listeria monocytogenes. *Nature Reviews Immunology*. **4:** 812–823
10. **Tsai YH, Disson O, Bierne H, Lecuit M.** 2013. Murinization of internalin extends its receptor repertoire, altering *Listeria monocytogenes* cell tropism and host responses. PLoS Pathog. **9:**e1003381.
11. **Wollert T, Pasche B, Rochon M, Deppenmeier S, van den Heuvel J, Gruber AD, Heinz DW, Lengeling A, Schubert WD.** 2007. Extending the host range of *Listeria monocytogenes* by rational protein design. Cell. **129:**891-902.
12. **Van Stelten, A.** (2014). Characterization of virulence-attenuated *Listeria monocytogenes* common among food and food-associated environments by rarely associated with disease. Doctoral dissertation, Texas Tech University. <https://ttu-ir.tdl.org/handle/2346/58916>
13. **Villanueva MS, Sijts AJ, Pamer EG.** 1995. Listeriolysin is processed efficiently into an MHC class I-associated epitope in *Listeria monocytogenes*-infected cells. J. Immunol*.* **155:**5227–5233.
